# Symmetry-enforced three-dimensional Dirac phononic crystals

**DOI:** 10.1038/s41377-020-0273-4

**Published:** 2020-03-10

**Authors:** Xiangxi Cai, Liping Ye, Chunyin Qiu, Meng Xiao, Rui Yu, Manzhu Ke, Zhengyou Liu

**Affiliations:** 1 Key Laboratory of Artificial Micro-Structures and Nano-Structures of Ministry of Education and School of Physics and Technology, Wuhan University, 430072 Wuhan, China; 2 Institute for Advanced Studies, Wuhan University, 430072 Wuhan, China

**Keywords:** Photoacoustics, Photonic devices

## Abstract

Dirac semimetals, the materials featuring fourfold degenerate Dirac points, are critical states of topologically distinct phases. Such gapless topological states have been accomplished by a band-inversion mechanism, in which the Dirac points can be annihilated pairwise by perturbations without changing the symmetry of the system. Here, we report an experimental observation of Dirac points that are enforced completely by the crystal symmetry using a nonsymmorphic three-dimensional phononic crystal. Intriguingly, our Dirac phononic crystal hosts four spiral topological surface states, in which the surface states of opposite helicities intersect gaplessly along certain momentum lines, as confirmed by additional surface measurements. The novel Dirac system may release new opportunities for studying elusive (pseudo) and offer a unique prototype platform for acoustic applications.

## Introduction

The discovery of new topological states of matter has become a vital goal in fundamental physics and material science^1,2^. A three-dimensional (3D) Dirac semimetal (DSM)^3–13^, accommodating many exotic transport properties such as anomalous magnetoresistance and ultrahigh mobility^14,15^, is an exceptional platform for exploring topological phase transitions and other novel topological quantum states. It is also of fundamental interest to serve as a solid-state realization of a (3 + 1)-dimensional Dirac vacuum. A DSM phase may appear accidentally at the quantum transition between normal and topological insulators^16,17^. The approach to such a single critical point demands the fine-tuning of the alloy’s chemical composition, which limits the experimental accessibility to the fascinating physics of 3D Dirac fermions. 3D DSMs can also emerge without fine-tuning parameters and are distinguished into two classes^3,4^. The first one, already realized in Na_3_Bi^7,8^ and Cd_3_As_2_^9,10^, occurs due to band inversion^5,6^. The Dirac points, lying on the generic momenta of a specific rotation symmetry axis, always come in pairs and could be eliminated by their merger and pairwise annihilation through the continuous tuning of parameters^3,4^ that preserve the symmetry of the system. The second class features Dirac points that are pinned stably to discrete high-symmetry points on the surface of the Brillouin zone (BZ). Markedly different from the first class of DSMs, the occurrence of Dirac points is an unavoidable result of the nonsymmorphic space group of the material^11–13^, which cannot be removed without changing the crystal symmetry. Although some solid-state candidate materials have been proposed^4,11,12^, symmetry-enforced 3D DSMs have never been experimentally realized because of the great challenge in synthesizing materials^4,7^.

Recently, numerous distinct topological states have been demonstrated in classical wave systems^18,19^, such as photonic crystals^20–28^ and phononic crystals^29–34^, which offer opportunities for exploring topological physics in a fully controllable manner. Here, we report an experimental realization of a 3D phononic crystal that hosts symmetry-enforced Dirac points at the BZ corners. The fourfold degeneracy is protected by a nonsymmorphic space group that couples point operations (rotations and mirrors) with nonprimitive lattice translations. In addition to the Dirac points identified directly by angle-resolved transmission measurements, highly intricate quad-helicoid surface states are unveiled by our surface measurements and associated Fourier spectra. Specifically, the surface states are composed of four gaplessly crossed spiral branches^13^ and thus are strikingly different than the double Fermi arc surface states observed recently in electronic^8^ and photonic systems^28^. Excellent agreement is found between our experiments and simulations.

**Fig. 1:**
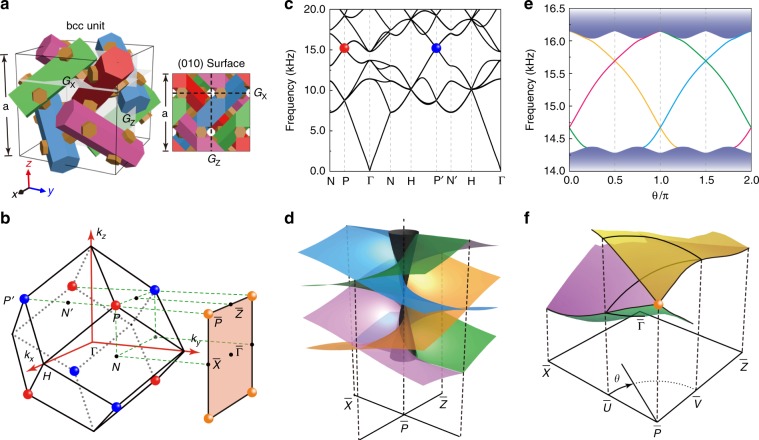
Symmetry-enforced Dirac points and quad-helicoid topological surface states in a nonsymmorphic phononic crystal. **a** Schematics of the bcc unit (left panel) of the phononic crystal and its (010) surface (right panel) featured with two glide mirrors *G*_*x*_ and *G*_*z*_. **b** 3D bcc BZ and its (010) surface BZ. The colored spheres highlight the bulk Dirac points with equal frequency and their projections onto the surface BZ. **c** Bulk bands simulated along several high-symmetry directions. **d** Schematic of the quad-helicoid surface state dispersions (color surfaces), where the gray cone labels the projection of bulk states. **e** Surface bands simulated along a circular momentum loop of radius 0.4*π*/*a* (as shown in **f**) centered at ${\bar{\mathrm P}}$. The shadow regions indicate the projected bulk states. **f** 3D plot of the surface dispersion simulated in the first quadrant of the surface BZ. Bulk band projections are not shown for clarity

As illustrated in Fig. 1a, our Dirac phononic crystal has a body-centered-cubic (bcc) lattice associated with the lattice constant *a* = 2.8 cm. The main body of the building block consists of four inequivalent resin cylinders, which are labeled with different colors and oriented along different bcc lattice vector directions. All the cylinders have a regular hexagonal cross section with a side length of 0.42 cm. To facilitate sample fabrication, these cylinders are connected with short hexagonal bars with side lengths of 0.21 cm. The remainder of the volume is filled with air. Numerically, the photosensitive resin material used for printing the acoustic structure is treated as rigid, and sound propagates only in air (at speed 342 m/s), considering the great acoustic impedance mismatch between the resin and air.

The crosslinked network structure belongs to the nonsymmorphic space group 230 $(Ia\bar 3d)$, featuring inversion symmetry and multiple screw rotations and glide reflections. The crystal symmetry enables rich point and line degeneracies (see Supplementary Materials). Interestingly, the small group at P and P’, a pair of time-reversal related Brillouin zone (BZ) corners (Fig. 1b), has 24 group elements and supports only fourfold degeneracy. This finding is confirmed by the band structure in Fig. 1c, which is stabilized with two distinct kinds of Dirac points at P (P’). The first kind of Dirac points, crossed with bands of different slopes and thus called generalized Dirac points^22^ (e.g., the lowest ones at P and P’ in Fig. 1c), corresponds to a four-dimensional irreducible representation, whereas the second kind, crossed with bands of identical slopes (see Fig. S1 in Supplementary Materials), corresponds to two inequivalent two-dimensional irreducible representations stuck with time-reversal symmetry. Hereafter, we focus on the latter case (as specified with color spheres in Fig. 1c), around which the bands are rather clean and carry a wide frequency window of linear dispersion. The system can be captured by a simple four-band effective Hamiltonian derived from $k \cdot p$ theory, $${\cal{H}} = \left( {\begin{array}{*{20}{c}} O & H \\ {H^\dagger } & O \end{array}} \right)$$, where $H = \eta \left( {\delta k_y\sigma _x - \delta k_x\sigma _y + \delta k_z\sigma _z} \right)$, *η* is a complex parameter determined by the acoustic structure, $(\delta k_x,\delta k_y,\delta k_z)$ characterizes the momentum deviation from P, and σ_*i*_ are Pauli matrices (see Supplementary Materials). The Dirac model gives isotropic linear dispersions around the Dirac point, which are much different from those anisotropic ones observed previously^7–10,28^. A nontrivial Z_2_ topological invariant, defined on a momentum sphere enclosing the Dirac points, can be used to depict the topology of such fourfold band closing points^13^. This invariant is derived by considering the pseudo anti-unitary symmetry ($\vartheta$) composited by a glide reflection (*G*) and time-reversal symmetry (*T*), i.e., $\vartheta = G \ast T$ with $\vartheta ^2 = - 1$. In addition, markedly different from the Dirac points created by band inversion^5,6^, which can be annihilated pairwise without changing the crystal symmetry, here the Dirac points are guaranteed completely by the nonsymmorphic symmetries. The topological robustness of the Dirac points against symmetry-preserving perturbations has been identified numerically by two detailed examples (Supplementary Materials, Fig. S2).

Unlike Weyl semimetals that host topologically nontrivial Fermi arcs on their surfaces^35^, the presence of topological surface states in a DSM is more subtle because the Dirac points carry a zero Chern number^3,4,36^. However, for a nonsymmorphic DSM that has Dirac points featuring a nontrivial Z_2_ index, the band crossing points will be pairwise connected by symmetry-protected Fermi arcs on the surface, associated with a unique connectivity determined by the nontrivial Z_2_ topological charge^13^. The dispersion of the topological surface states can be mapped to an intersecting multihelicoid structure, where the intersections between the helicoids are protected from being gapped by the glide symmetries preserved on the specific surface. In our case, the Dirac phononic crystal supports elusive quad-helicoid surface states^13^ if truncated with the (010) surface or its equivalents, which can be characterized by the wallpaper group *p*2*gg*. Below, we focus on the (010) surface that preserves the two glide mirrors $G_x = \left\{ {M_z\left| {\left( {a/2} \right)\hat x + \left( {a/2} \right)\hat z} \right.} \right\}$ and $G_z = \left\{ {M_x\left| {\left( {a/2} \right)\hat z} \right.} \right\}$ of the bulk crystal (Fig. 1a). For this specific crystal surface, the two inequivalent Dirac points are projected onto the four *equivalent* surface BZ corners ${\bar{\mathrm P}}$ (Fig. 1b). As schematically illustrated in Fig. 1d, the quad-helicoid surface states feature two crucial signatures. First, there are four branches of spiral surface states for any given momentum loop enclosing ${\bar{\mathrm P}}$: two with positive helicities and two with negative helicities. Figure 1e shows the gapless surface bands simulated along a circular loop centered at ${\bar{\mathrm P}}$. Second, the surface states of opposite helicities intersect along certain momentum lines, in which the intersecting double degeneracies are protected by the glides *G*_*x*_ and *G*_*z*_ assisted with time-reversal symmetry^13^. This effect is exhibited clearly in the simulated global dispersion profile (Fig. 1f), which shows nodal line degeneracies along the surface BZ boundaries ${\bar{\mathrm {P}}}{\bar{\mathrm {X}}}$ and ${\bar{\mathrm {P}}}{\bar{\mathrm {Z}}}$. (Only ¼ of the surface BZ is provided due to the presence of the two glides.) For a generic selection of the crystal surface, the nodal line degeneracy of the surface dispersion disappears due to the absence of glide symmetries (Supplementary Materials, Fig. S3).

**Fig. 2:**
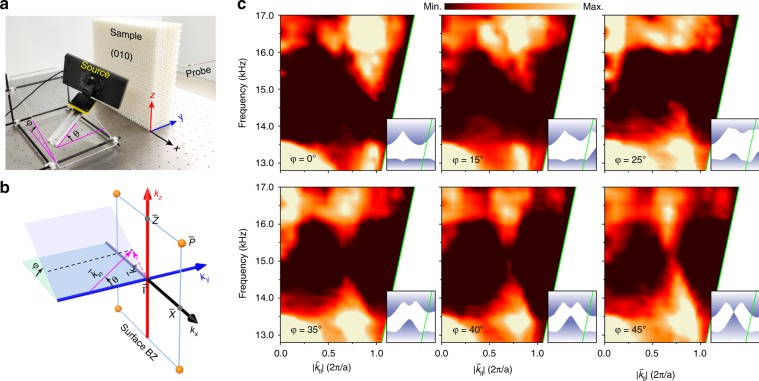
Experimental identification of the symmetry-enforced Dirac points. **a** Experimental setup for measuring sound transmission. **b** Schematic of exciting bulk states according to the momentum conservation $\vec{k} _{||} =\vec{k} _{in}{\rm{sin}} \theta$. **c** *θ*-resolved transmission spectra measured for different *φ* values. The slanted boundary (green line) in each panel corresponds to the ‘sound cone’ $|\vec{k} _{||}| = |\vec{k} _{in}|$, beyond which no transmission can be measured. Insets: Simulated bulk states (shadow regions) projected along the *y* direction, scaled to the same range and ratio as the measured data

The presence of symmetry-enforced Dirac points was confirmed by angle-resolved transmission measurements. Figure 2a demonstrates our experimental setup. The sample, fabricated precisely by the 3D printing technique, has a size of 47.6 cm, 14.0 cm, and 47.6 cm along the *x*, *y,* and *z* directions, respectively. A rectangular acoustic horn was used to launch a collimated beam upon the (010) surface of the sample, where the incident direction can be characterized by the angles *θ* and *φ*. As illustrated in Fig. 2b, a bulk state is expected to be excited when its in-plane momentum $\vec{k} _{||}$ matches that of the incident wavevector $\vec{k} _{in}\sin \theta$ at the same frequency. The transmitted sound signal was scanned by a 1/4 inch microphone (B&K Type 4958-A) and recorded by a multi-analyzer system (B&K Type 3560B). The averaged sound intensities were normalized to those measured in the absence of the sample. The bulk states were mapped out by varying *θ* and *φ*. Here, only $\varphi \in [0,45^\circ ]$ was focused thanks to the multiple glide mirrors of the system. (For completeness, similar data for $\varphi \in [45^\circ ,90^\circ ]$ are provided in Supplementary Materials, Fig. S4.) Specifically, at *φ* = 45°, the incident beam scans through the Dirac point. Figure 2c shows the transmission data measured for six representative *φ* values compared with the numerical bulk dispersions projected along the corresponding directions (insets). All the transmission spectra agree reasonably well with the numerical band structures, where the low transmission near the sound cone can be attributed to the smaller effective cross-sectional area of the sample at large *θ*. In particular, as expected in the case of *φ* = 45°, a conic touch is observed at approximately 15.3 kHz in frequency and 0.71*π*/*a* in the wavevector. The point crossing is lifted gradually as *φ* decreases from 45°.

**Fig. 3:**
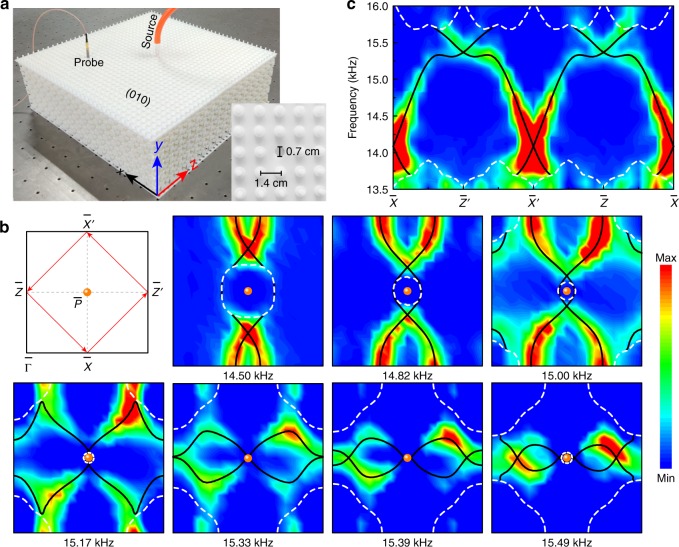
Experimental observation of quad-helicoid topological surface states. **a** Experimental setup for the surface field measurements. The inset shows the details of the cover plate with circular holes opened or sealed. The plugs that sealed the holes were opened one-by-one during the measurement. **b** Isofrequency contours plotted in one surface BZ centered at ${\bar{\mathrm P}}$ (see the first panel). The color scale shows the experimental data compared with the corresponding simulation results (black curves). The orange spheres label the projected Dirac points, and the white dashed lines enclose the bulk band projections. **c** Frequency-dependent surface spectra (color scale) measured along the momentum path specified in the first panel of **b**

Furthermore, we performed surface measurements to identify the highly intricate topological quad-helicoid surface states, which have not been experimentally observed in any topological system to date. Figure 3a shows our experimental setup. To mimic the rigid boundary condition involved in our simulations, an additional resin plate with a thickness of 0.2 cm was integrated on the (010) surface, which served as a trivial acoustic insulator to guarantee the presence of topological surface states. Since the typical air channels of the sample are too narrow to accommodate the sound source and probe directly, the plate was perforated with a square lattice of holes (see inset), one of which was reserved for inserting sound source, and one of which was reserved for locating the probe during the measurement; the other holes not in use were sealed to avoid coupling with the air background surrounding the sample. To excite surface states, a broadband point-like sound source launched from a subwavelength-sized tube was injected into one hole near the center of the sample surface. The localized surface field was scanned hole-by-hole by manually moving the probe, where the scanning step was given by the lattice spacing of the holes (1.4 cm). By Fourier transforming the surface pressure field, we mapped out the nontrivial surface arc for any desired frequency^31^. Figure 3b shows such data for a sequence of frequencies. As predicted by the simulations, the measured surface arcs (bright color) exhibit clear crossings at the surface BZ boundaries ${\bar{\mathrm {X}}}{\bar{\mathrm {X}}}^{\prime}$ and ${\bar{\mathrm {Z}}}{\bar{\mathrm {Z}}}^{\prime}$. Our experimental results effectively capture the simulated isofrequency contours of the topological surface states (black lines), despite the band broadening due to the finite-size effect. Note that the amplitude signals of the bulk states (enclosed by white dashed lines) are much weaker than those of the topological surface states that are highly confined to the surface. To further identify the gapless quad-helicoid surface states, we present the surface spectra (Fig. 3c) measured along the momentum loop specified in the first panel of Fig. 3b. Compared with the loop used in Fig. 1e, this square loop enclosing the ${\bar{\mathrm P}}$ point is larger and favored to demonstrate the gapless intersection of the surface bands in a wide bulk gap. As expected, two pairs of surface bands with opposite helicities traverse the bulk gap and cross stably at the high-symmetry momenta ${\bar{\mathrm X}}$ (${\bar{\mathrm {X}}}^{\prime}$) and ${\bar{\mathrm Z}}$ (${\bar{\mathrm {Z}}}^{\prime}$). Again, excellent agreement is found between our experiment and simulation.

In conclusion, we have constructed and identified a spinless Dirac crystal working for airborne sound, which exhibits highly intricate properties in both the bulk and surface states, in sharp contrast to those realized previously in condensed matter systems^7–10^. The topological origin of quad-helicoid surface states deserves to be further investigated. Notably, in a very recent study, S. Zhang et al. have made the first step towards an experimental study of 3D Dirac points in classical wave systems^28^. Interestingly, the Dirac points are constructed by electromagnetic duality symmetry (which is unique in electromagnetic systems), which is also strikingly different from the crystalline symmetry involved here. Starting with our structure, one can design various interesting 3D acoustic topological states (e.g., Weyl points^29,31^ and line nodes^37,38^) through symmetry reduction. This study may open up new manners for controlling sound, such as realizing unusual sound scattering and radiation, considering the conical dispersion and vanishing density of states around the Dirac points. Last but not least, the dispersion around the Dirac point is isotropic, and thus, our macroscopic system serves as a good platform to simulate relativistic Dirac physics.

## Methods

### Numerical simulations

All simulations were performed using COMSOL Multiphysics, a commercial solver package based on the finite element method. The bulk band structure in Fig. 1c was calculated by a single unit cell imposed with specific Bloch boundary conditions. Similar calculations gave the projected bulk states along the *y* direction (Fig. 2c, shadowed region). A ribbon structure was used to calculate the surface band for a desired surface (Fig. 1e, f and Fig. 3b, c), imposed with Bloch boundary conditions along the *x* and *z* directions and a rigid boundary condition along the *y* direction, respectively. The ribbon was long enough to avoid coupling between the opposite surfaces. Surface states were distinguished from the projected bulk states by inspecting the surface localizations of the eigenstates.

### Experimental measurements

Our experiments were performed for airborne sounds at audible frequencies. The slab-like sample, consisting of 17 × 5 × 17 structural units along the *x*, *y,* and *z* directions, was prepared by photosensitive resin via 3D printing. The macroscopic characteristics of our acoustic system enable precise sample fabrication and less demanding signal detection. To excite the bulk states, a rectangular acoustic horn (with a surface area of 24.0 cm × 10.0 cm) was used to launch Gaussian beams at controllable orientations (Fig. 2a), whereas a narrow tube (with a diameter of 0.8 cm) was used to export point-like sound signals to excite the topological surface states (Fig. 3a). During both measurements, a portable microphone was moved on the *x–z* plane to scan the pressure fields, together with another identical microphone fixed for phase reference. Both the amplitude and phase information of the input and output signals, swept from 11.8 kHz to 18.2 kHz with an increment of 0.032 kHz, were recorded and analyzed by a multi-analyzer system. To map out each surface arc of a given frequency (Fig. 3b), two-dimensional Fourier transformation was performed on the scanned surface field; this further gave the frequency-dependent surface spectra along the specific momentum loop (Fig. 3c).

## Supplementary information

MATERIAL Supplementary information for Symmetry-enforced three-dimensional Dirac phononic crystals

## Data Availability

The data that support the plots within this paper and other findings of this study are available from the corresponding author upon reasonable request.

## Code availability

All codes that support this study are available from the corresponding author upon reasonable request.
