# Supplementary material for: Symmetry-enforced three-dimensional Dirac phononic crystals
Source: Light Sci Appl. 2020 Mar 10;9:38. doi: 10.1038/s41377-020-0273-4 (PMC7064592; doi:10.1038/s41377-020-0273-4)
Supplement: Supplementary file 1 — MATERIAL Supplementary information for Symmetry-enforced three-dimensional Dirac phononic crystals [file 41377_2020_273_MOESM1_ESM.docx]

Supplementary information for

**“Symmetry-enforced three-dimensional Dirac phononic crystals”**

Xiangxi Cai,^1†^ Liping Ye,^1*^ Chunyin Qiu,^1^ Meng Xiao,^1^ Rui Yu,^1^ Manzhu Ke,^1^

Zhengyou Liu^1,2*^

^1^Key Laboratory of Artificial Micro- and Nano-structures of Ministry of Education and School of Physics and Technology, Wuhan University, Wuhan 430072, China ^2^Institute for Advanced Studies, Wuhan University, Wuhan 430072, China

^*^Correspondence to: [lpye@whu.edu.cn;](mailto:lpye@whu.edu.cn;) [zyliu@whu.edu.cn](mailto:zyliu@whu.edu.cn)

1. **Symmetry-protected band degeneracies along the high-symmetry directions**

As shown in Fig. 1 in the main text, the nonsymmorphic space group 230 ($\mathrm{Ia}\bar{3}d$) of the phononic crystal enables rich degeneracies along the high-symmetry momentum lines. Below we list the little groups of those momentum lines to interpret such degeneracies.

(a) $\mathrm{NP}$ direction: $\left\{ E,\left\{ 2_{001} | 0\frac{1}{2}0 \right\},\left\{ m_{110} | \frac{3}{4}\frac{1}{4}\frac{1}{4} \right\},\left\{ m_{1\bar{1}0} | \frac{1}{4}\frac{1}{4}\frac{1}{4} \right\} \right\}$.

It supports one two-dimensional (2D) irreducible representation and contributes only double degeneracy along this momentum line. A similar result holds for $P'N'$.

(b) $P\Gamma$ direction: $\left\{ E,\left\{ 3_{111}^{+} | 000 \right\},\left\{ 3_{111}^{-} | 000 \right\},\left\{ m_{1\bar{1}0} | \frac{1}{4}\frac{1}{4}\frac{1}{4} \right\},\left\{ m_{01\bar{1}} | \frac{1}{4}\frac{1}{4}\frac{1}{4} \right\},\left\{ m_{\bar{1}01} | \frac{1}{4}\frac{1}{4}\frac{1}{4} \right\} \right\}$.

It supports two one-dimensional (1D) representations plus one 2D irreducible representation, and thus contributes single bands and doubly degenerate bands simultaneously. A similar result holds for $HP'$.

(c) $\Gamma N$ direction: $\left\{ E,\left\{ 2_{110} | \frac{3}{4}\frac{1}{4}\frac{1}{4} \right\},\left\{ m_{001} | 0\frac{1}{2}0 \right\},\left\{ m_{1\bar{1}0} | \frac{1}{4}\frac{1}{4}\frac{1}{4} \right\} \right\}$.

It supports four 1D representations and contributes only single bands.

(d) $\mathrm{NH}$ direction: $\left\{ E,\left\{ 2_{1\bar{1}0} | \frac{1}{4}\frac{1}{4}\frac{1}{4} \right\},\left\{ m_{001} | 0\frac{1}{2}0 \right\},\left\{ m_{110} | \frac{3}{4}\frac{1}{4}\frac{1}{4} \right\} \right\}$.

It supports one 2D irreducible representation and contributes only doubly degenerate bands. A similar result holds for $N'H$.

(e) $H\Gamma$ direction:

It supports four 1D representations and one 2D irreducible representation, and thus contributes single bands and doubly degenerate bands simultaneously.

1. **Effective Hamiltonian in the vicinity of $\mathbf{P}$ point**

Based on the $k\cdot p$ theory, below we derive the effective Hamiltonian $\mathcal{H}$ in the vicinity of $P$ point. At this momentum the little group $G_{P}$ has 24 group elements and supports two four-dimensional (4D) representations: one is 4D irreducible, and the other is stuck by a pair of inequivalent 2D irreducible representations under the assistance of time-reversal symmetry.

Near the fourfold degenerate point $P$, the phononic crystal can be described by the eigen problem

${\mathcal{H}_{ij}U}_{j}=2\omega_{P}\delta\omega U_{i}$, , (S1)

where $\omega_{P}$ represents the frequency of $P$ point, and $\delta\omega$ and characterize the frequency and momentum deviations from $P$ [see Phys. Rev. B **89**, 134302 (2014)]. The vector matrix , in which and are periodically distributed bulk modulus and mass density of the sound media, respectively. The base functions $\left. \left| \psi_{i} \right. \right\rangle$ satisfy the orthogonal relationship $\left\langle\psi_{i} | \psi_{j} \right\rangle=\delta_{ij}$, where $\delta_{ij}$ is Kronecker Delta and the integral is done over the whole primitive cell. It is easy to derive for the real sound parameters (i.e., ignoring absorption). The form of the effective Hamiltonian $\mathcal{H}$ can be simplified through the constraints imposed by the symmetry of the phononic crystal,

$D_{li}^{*}D_{mj}R_{nk}^{*}\mathfrak{p}_{lm,n}=\mathfrak{p}_{ij,k}$, (S2)

where $D\left( g \right)$ is the little representation of the group element $g\in G_{P}$ under the base functions $\left\{ \psi_{i} \right\}$, and $R(g)$ is the representation of $g$ under the bases of Euclidean space $\left\{ \hat{x}_{i} \right\}$. Only the four generators of $G_{P}$ are required to be taken into account, i.e., $\left\{ 3_{\bar{1}\bar{1}1}^{+} | \frac{1}{2},0,0 \right\}$, $\left\{ 2_{010} | \frac{1}{2},0,0 \right\}$, $\left\{ 2_{100} | 0,0,\frac{1}{2} \right\}$, and $\left\{ 4_{100}^{-} | \frac{3}{4},\frac{1}{4},\frac{1}{4} \right\}$. Details for the representations *D* and *R* are listed in Table 1, where $D_{4}$ denotes the 4D irreducible representation, and $D_{2\bigoplus2}$ denotes the direct sum of two inequivalent 2D irreducible representations.

*Case I: Anisotropic Dirac Point.* Substituting the explicit expressions of $D_{4}$ and $R$ into Eq. (S2), relationships among the matrix elements $\mathfrak{p}_{ij,k}$ can be established. Finally, the vector matrix involves only two independent elements $\mathfrak{p}_{12,y}=\alpha$ and $\mathfrak{p}_{13,x}=\beta$, both of which are real numbers determined by the phononic crystal structure. The effective Hamiltonian can be written as

$\mathcal{H=}\left( \begin{matrix} H_{0} & H_{I} \\ H_{I}^{\dagger} & H_{0} \end{matrix} \right)$. (S3)

Here $H_{I}=\frac{\beta}{\sqrt{2}}ⅇ^{ⅈ\frac{\pi}{12}}\left\{ {ⅇ^{ⅈ\frac{\pi}{6}}\delta k_{z}\sigma_{0}-\delta k_{x}\sigma_{x}+ⅇ}^{ⅈ\frac{\pi}{3}}\delta k_{y}\sigma_{x}+\delta k_{x}\sigma_{y}+ⅇ^{ⅈ\frac{\pi}{3}}{\delta k_{y}\sigma}_{y}+ⅇ^{-ⅈ\frac{\pi}{3}}{\delta k_{z}\sigma}_{z} \right\}$ and $H_{0}=\alpha\left( \delta k_{y}\sigma_{x}-{\delta k}_{x}\sigma_{y}+\delta k_{z}\sigma_{z} \right)$, with $\sigma_{i}$ being Pauli matrices. In general, this (Hermitian) Hamiltonian gives four completely independent (nonzero) eigenvalues, and the dispersions around the degenerate point $P$ are linear and anisotropic. Specifically, in Fig. 1c, this kind of (generalized) Dirac points occurs at the *unlabeled* fourfold crossing points. The aforementioned properties can be seen clearly from the slopes of the dispersions around $P$. For example, we consider the Dirac point with the lowest frequency: along the $\mathrm{NP}$ direction, two degenerate bands have negative slopes and two degenerate bands have positive slopes; along the $P\Gamma$ direction, however, the first band has a negative slope and the rest three bands (two of which are degenerate) have positive slopes.

*Case II: Isotropic Dirac Point.* Substituting the explicit expressions of $D_{2\bigoplus2}$ and $R$ into Eq. (S2), we find that the matrix involves only one independent complex number $\eta=\mathfrak{p}_{13,z}$, and the effective Hamiltonian can be simply written as

$\mathcal{H=}\left( \begin{matrix} O & H \\ H^{\dagger} & O \end{matrix} \right)$, (S4)

associated with $H={\eta(\delta k}_{y}\sigma_{x}{-\delta k}_{x}\sigma_{y}+\delta k_{z}\sigma_{z})$. The eigenvalues of $\mathcal{H}$, , provide directly the *isotropic* slopes for the linear dispersions around the point $P$. (This has been identified in Fig. S1 below.) Specifically, in Fig. 1c, the degenerate points labeled with color spheres belong to this kind of Dirac points, which are crossed with two sets of doubly degenerate bands in the specific directions.


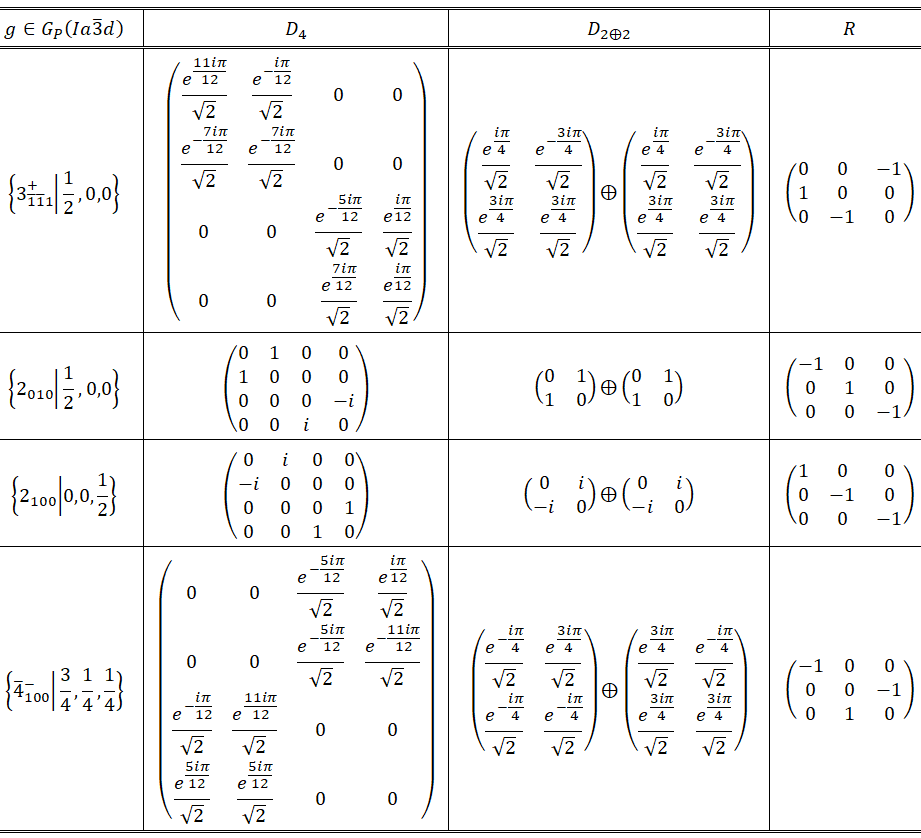


**Table S1.** Detailed forms of the representations $D$ and $R$ for the four generators of the little group $G_{P}$, where $D_{4}$ denotes the 4D irreducible representation, and $D_{2\bigoplus2}$ denotes the direct sum of two inequivalent 2D irreducible representations.

**
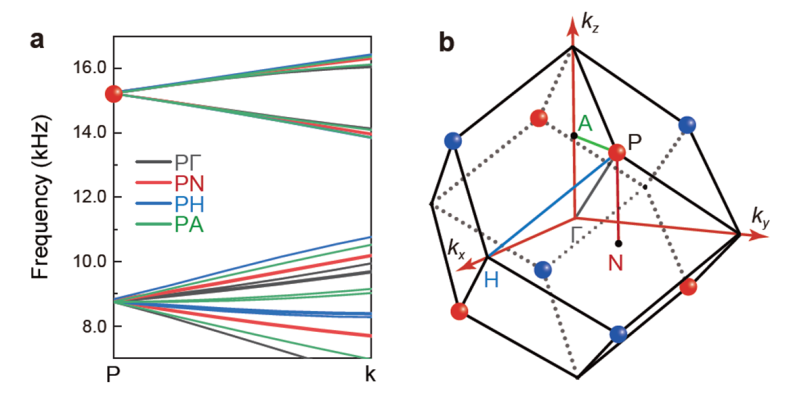
**

**Figure S1 | Slope information of the** **bulk dispersions around the point P. a**, Bulk dispersions plotted along the four momentum lines labeled in the bulk BZ in **b**. As predicted by the k.p theory, the band slopes around the higher-frequency crossing points are identical, in contrast to those bands around the lower-frequency crossing point.


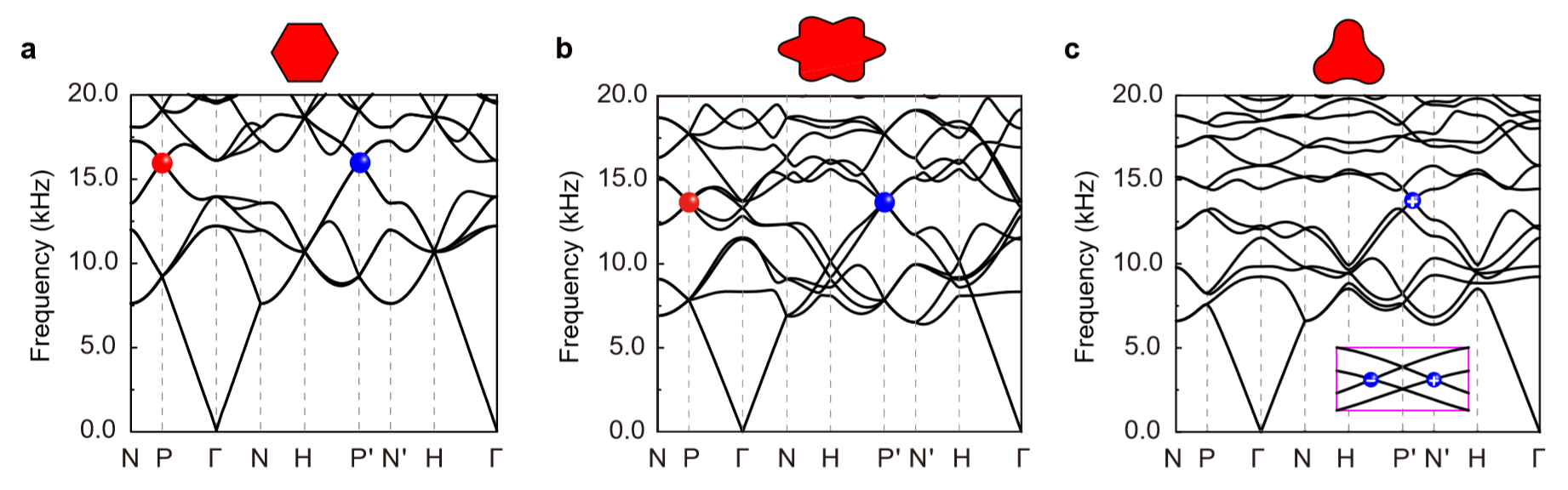


**Figure S2 |** **Numerical examples that identify the topological robustness of the Dirac points against the symmetry-preserving/breaking perturbations.** The red patterns above the dispersions give the corresponding cross section of the main cylinders. **a**, Bulk dispersion calculated for a structure resembling Fig. 1a, in which the short bars connecting the hexagonal cylinders are removed. It shows that the Dirac points (color spheres) survive under such a symmetry-preserving perturbation. **b**, Bulk dispersion calculated for a structure resembling Fig. 1a, but the cross section of the main cylinder is deformed to sacrifice the threefold rotation. In this case, the acoustic structure loses the threefold screw symmetries and thus belongs to the nonsymmorphic space group 73 ($\mathrm{Iabc}$). As shown in Fig. S1b, the Dirac points survive at $P$ and $P'$. Note that this structure has the lowest crystalline symmetry to maintain the symmetry-enforced Dirac points in the bcc lattice. **c**, Bulk dispersion calculated for a structure resembling Fig. 1a, while the hexagonal cylinders are changed into triangular ones. This leads to the breaking of the glide reflection $G_{y}$, and the whole structure belongs to the nonsymmorphic space group 45 ($Ib2a$). As a supplement, the inset provides an amplified dispersion (centered at $P'$) along the $PP'$ direction. We can see, each Dirac point splits into a pair of oppositely charged Weyl points if one of the glide symmetries is broken.


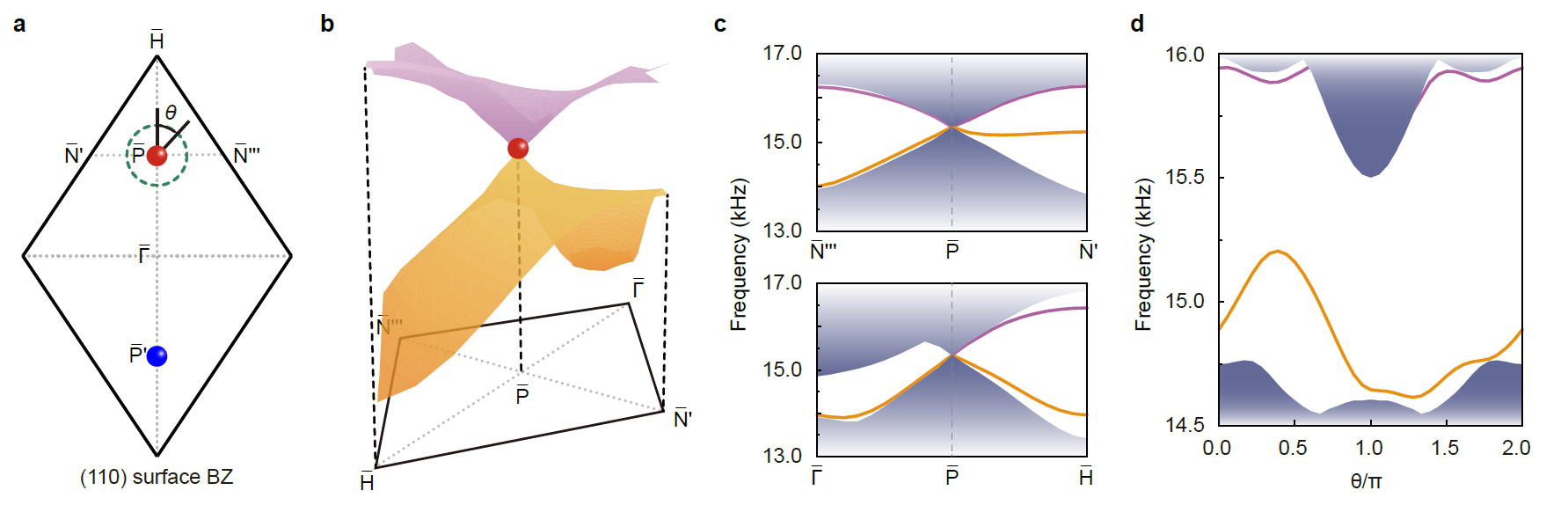


**Figure S3 | Surface states for a generic selection of the crystal surface, such as (110). a**, One surface BZ for (110). The red and blue spheres at $\bar{\text{P}}$ and $\bar{\text{P}}'$ indicate the projections from the inequivalent bulk Dirac points at P and $\text{P}'$, respectively. **b**, 3D plot of the surface dispersion around the projection $\bar{\text{P}}$. Bulk band projections are not shown for clarity. **c**, Surface dispersions simulated along two specific directions. The shadow regions indicate the projected bulk states. **d**, Surface dispersion simulated along the circular momentum loop specified in **a**, which encircles the projection of a single Dirac point. Much different from the case of (010) surface, doubly degenerate linear crossings disappear in this case since there is no additional glide symmetry to protect them. Similar behavior occurs for a more generic crystal surface.


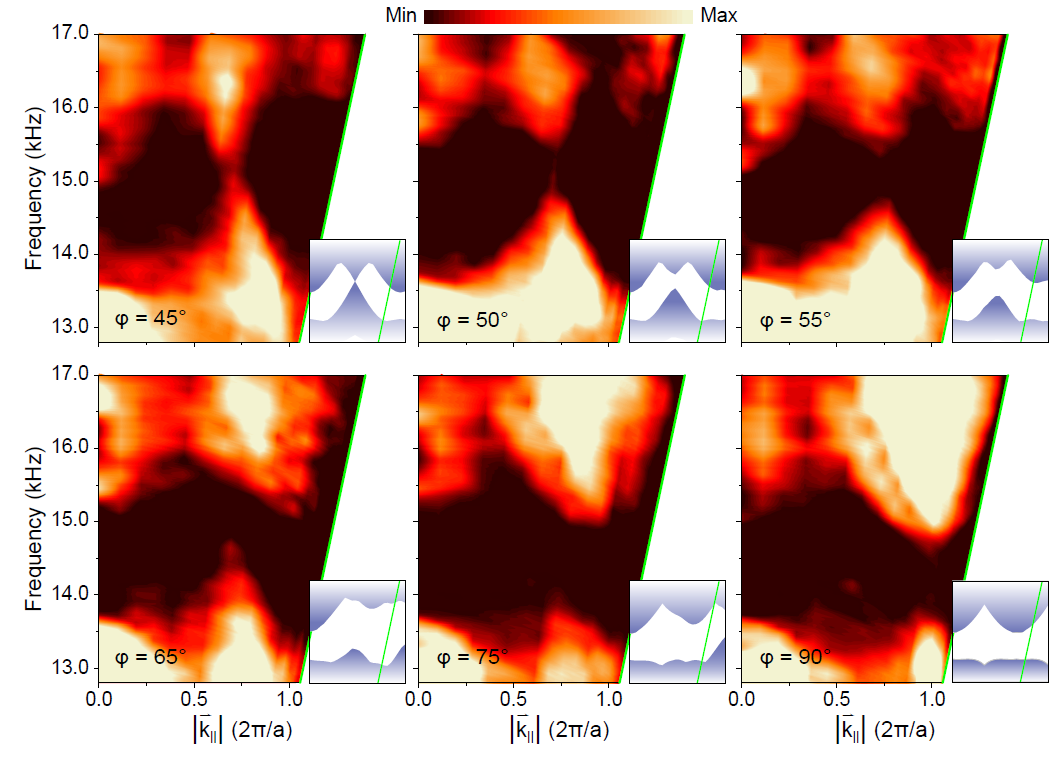


**Figure S4 | $\theta$-resolved transmission spectra measured for , similar to Fig. 2c**. The slanted boundary (green line) in each panel corresponds to the ‘sound cone’ $\text{|}\vec{k}_{||}\text{|}=|\vec{k}_{\text{in}}|$, beyond which no transmission can be measured. Insets: Simulated bulk states (shadow regions) projected along the *y* direction, scaled to the same range and ratio as the measured data. As expected, the point crossing is lifted gradually as grows from $45^{\circ}$. All the transmission spectra agree reasonably well with the numerical band structures.


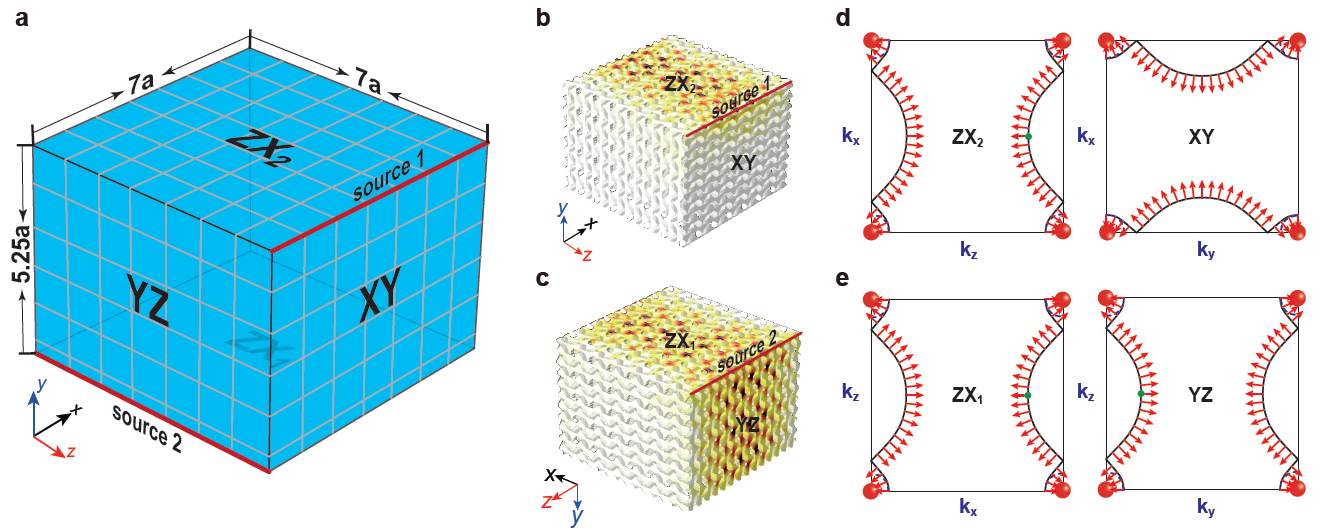


**Figure S5 | Numerical examples for illustrating the properties of surface states. a**, Sample geometry and its surface details. The sample has a dimension of 7*a*, 5.25*a* and 7*a* along the *x*, *y* and *z* directions, respectively. Each 3D grid indicates a perfect unit cell plotted in Fig. 1 (main text). As such, all the surfaces exhibit identical geometric details, except the bottom surface (ZX_1_). In each simulation, we position a line source (red line) at one of the sample hinges, which effectively launch Gaussian beams towards the two surfaces sharing the hinge. **b** and **c**, Simulated pressure amplitude profiles (at 14.82 kHz) for the case of source 1 and source 2. The simulations exhibit much different behaviors: the source 1 ignites only the ZX_2_ surface but the source 2 excites both the ZX_1_ and YZ surfaces. **d** and **e**, Isofrequency contours at 14.82 kHz (black lines) for the associated four surfaces, which interpret the above phenomena. The red arrows point to the group velocity of the surface states. The green point labeled on the isofrequency contour correspond exactly to the surface state excited by the acoustic line source, associated with nearly zero momentum component parallel to the hinge.
